# Research on key technology of cooled infrared bionic compound eye camera based on small lens array

**DOI:** 10.1038/s41598-024-61606-1

**Published:** 2024-05-15

**Authors:** Xiaoyu Wang, Linhan Li, Yinghao Chi, Jie Liu, Juan Yue, Sili Gao, Xiupeng Yuan, Yang Yu

**Affiliations:** 1 Shanghai Institute of Technical Physics, Chinese Academy of Sciences, Shanghai, 200028 China; 2 University of Chinese Academy of Sciences, Beijing, 100864 China; 3 Key Laboratory of Infrared Detection and Imaging Technology, Chinese Academy of Sciences, Shanghai, 200028 China

**Keywords:** Engineering, Optics and photonics, Physics

## Abstract

Traditional 2D imaging technologies are limited by the need for a large field of view and their sensitivity to small target motion. Inspired by the characteristics of insect compound eye structure, we propose an infrared bionic compound eye camera based on a small lens array. The camera is composed of 61 small lens arrays mounted on a curved spherical shell and a relay optical system. The imaging device is a high-performance cooled mid-wave infrared detector. This is an innovative design for an infrared biomimetic compound eye camera system that provides a wide field of view and all-day detection capability. Aimed to meet the specified requirements. The optical system achieves a 100% cold-membrane match between the infrared optical system and the cooled detector, and the relay optical system optimizes the large-field aberration by introducing a higher-order aspheric surface and modifying the geometric surface of the lenses. Our entire system enables an observation field angle of $108^\circ \times 108^\circ$. The experiments showed that the image quality of the system is high, each ommatidium was effective within the imaging range of the compound eye camera, resulting in an improved signal-to-noise ratio in various scenes.

## Introduction

Over 500 million years of evolution, arthropods have developed complex compound eyes with special visual capabilities^1^. In nature, common dragonflies, fruit flies, ants, beetles, and bees have a pair of hemispherical compound eyes made up of many tiny optical units. The field of view of these insect compound eyes is typically larger than that of the human eye, and when observing a target at a specific distance, the compound eye system has the advantage of being able to quickly search for and acquire information about the target while at rest. Based on these claims, researchers have continued to push the development of artificial compound eyes. With the continuous development of three-dimensional imaging technology, people’s demands for imaging technology have continued to improve, and the application of various imaging technologies has become more widespread. It is not surprising to draw inspiration from biomimetics, as the advent of fisheye lenses and wide-angle lenses has facilitated the acquisition of wide-field scene information. However, the high distortion rates at the edges of fisheye and various wide-angle lens imaging systems significantly reduce the accuracy and credibility of scene information capture. Compound-eye cameras, on the other hand, exhibit rapid imaging response and reduced edge distortion, yet they have lower imaging resolution^2^. In light of the numerous imaging characteristics of the compound-eye camera, this article innovatively proposes a compound-eye imaging system based on a cryogenic mid-wave infrared detector. This system addresses the lack of night vision capability in visible light cameras and the issue of significant edge distortion in traditional wide-field single-aperture cameras. It provides the possibility for all-day large-field target detection and rapid localization of stationary targets.

**Figure 1:**
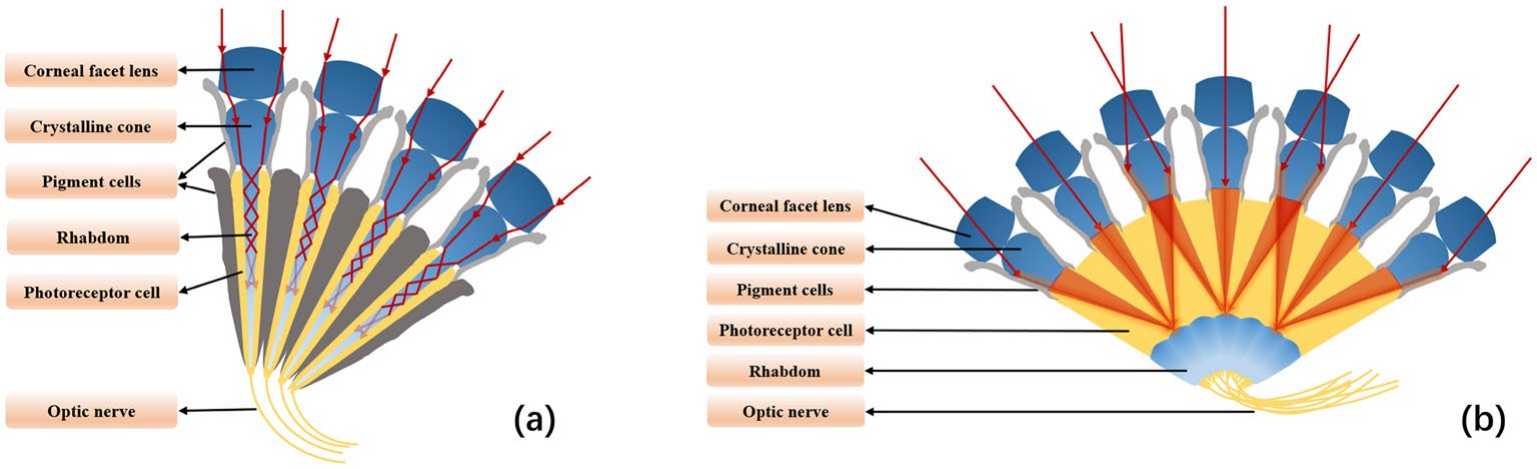
Insect compound eye structures in two main classifications. (**a**) Juxtaposed structure. (**b**) Overlapping type structure.

The compound eye of an insect, consisting of a corneal lens, lens cones, and sensory rod bundles, is divided into overlapping and juxtaposed structures. In the juxtaposed structure shown in Fig. 1a, each ommatidium unit is relatively independent and corresponds to a limited single visual field, and the incident light passes through the corneal lens to be received by the sensory rod bundles of the ommatidia, which are then transmitted by the optic nerve to the brain for processing^3,4^. There is an overlap of the visual fields of the ommatidia in the compound eye of the overlapping type structure shown in Fig. 1b , the distinguishing feature is that each ommatidium is not a separate unit but is interconnected. According to its internal structure, it can be further classified into refractive, and parabolic overlapping. Overlapping compound eyes have a special ability to adapt and are more sensitive to low light. Light enters through the cone side of the cornea. The neighboring ommatidia provide more light. Beneath the fovea is a dense layer of photoreceptor cells (sensory rod bundles) that convert light into electrical signals that are sent to the brain. Due to the characteristics of large field of view, high sensitivity, and high sensitivity in biological compound eyes, various industries have successively carried out a series of research and applications on artificial compound-eye imaging systems. These include wide-field imaging, high-resolution imaging, target distance detection, medical imaging, self-motion estimation, and navigation. In previous studies, researchers mainly focused on two types of biomimetic compound eye structures: one based on micro-lens arrays and the other based on small lens arrays for compound-eye imaging systems.

With respect to the design of compound eye microlens cameras, a Japanese research group proposed the TOMBO (Thin Observation Module by Bound Optics) compound eye imaging system for the design of a microlens compound eye camera in 2000. The system comprises an optical signal acquisition layer with a microlens array, an optical isolation layer, and a photodetector assembly. Each microlens and its corresponding CMOS sensor form a single imaging unit^5,6^. In 2004, a German research group proposed the APCO (Artificial Apposition Compound Eye Objective) artificial compound eye imaging system. Its structure is similar to TOMBO, consisting of a planar microlens array matched with a metal aperture counterpart, capable of realizing a multi-channel imaging function^7–9^. In 2014, Robert Leitel et al. proposed the introduction of curvature to enhance the field of view of the artificial compound eye sensor to $180^\circ \times 60^\circ$, and the integration of photoreceptor arrays based on optical flow detection with compound eye optics to improve sensitivity and expand the field of view, facilitating the navigation of the optical flow calculations^10^. In 2017, KUO PANG et al. developed a single-layer compound eye structure with seven ommatidia. The functional relationship between the field of view, optical axis angle, and overlap area was mathematically analyzed, and related algorithms such as the Kalman filter were applied to achieve high-speed target capture for 3D tracking^11^. In 2020, Zhou Peilin et al. fabricated an artificial compound eye with waterproof properties inspired by the natural superhydrophobic surface of lotus leaves. They used a special method to prepare nano lens arrays to make the flexible membrane exhibit superhydrophobic properties, and the deformation of the MLAs membrane produced an artificial compound eye with a variable FOV ranging from $0^\circ$ to $160^\circ$^12^. 2021 Dai et al. created bionic appositional compound eyes using microfluidic-assisted 3D printing. Each micro-lens is connected to the bottom plane of the eye through a zero-crosstalk refractive index-matched waveguide to mimic the transverse striations of the natural eye to achieve full-color wide-angle panoramic views and point source position tracking^1^. In 2022, Zhi-Yong Hu et al. used femtosecond laser two-photon polymerization to fabricate a polymer compound eye with 19-160 log-profile ommatidia, which greatly increases the camera’s depth-of-field and focusing range^13^. 2024 Dewen Cheng et al. fabricated sub-lenses based on a three-layer structure and discrete arrangement to suppress stray light and enable spatial refocusing methods to recover image information at different object depths. This system is well-suited for close-up photography applications that require short conjugate distances and small device sizes. It solves the problem of crosstalk between neighboring channels^14^. In 2024, Kenji Yamada et al. proposed a new system that integrates pulse wave detection and personal identity authentication using compound eye optics. A robust, efficient, and non-invasive method for measuring pulse waves and identifying individuals is provided^15^.

In terms of lens array camera design, in 2012, Brady et al. described the AWARE-2 camera, which has a single aperture with a diameter of 16 mm and can capture 1 gigapixel images at three frames per minute, and AWARE-2 solves the problem by using a parallel microlens array that converts gigapixel images to megapixel images^16^. In 2015, Cao et al.proposed a design method for a spherical compound eye consisting of 37 imaging channels. This device is composed of multidimensional sub-eye imaging channels from different observation directions and combined with segmented rotating projection technology for corresponding imaging experiments. The experiments verified its observation field reaching up to $118^\circ$^17^. In 2017, Shi CY et al. demonstrated a three-part hemispheric compound eye, an optical relay system, and a commercial CMOS imaging sensor hemispheric compound eye camera (SCECam), which can achieve a large observation field of view of up to $122.4^\circ$ and can detect and locate the speed of fast-moving objects^18^.In 2023, Zhang Yuanjie and colleagues proposed a novel biomimetic multispectral curved-eye camera (BM3C), capable of achieving a maximum field of view (FOV) of $120^\circ$ and capturing seven-band multispectral images from the visible light to near-infrared bands. They conducted aerial imaging experiments using drones and performed land-type classification using the K-means method. The experiments demonstrated the significant application value of the BM3C imaging system in remote sensing for aerial imaging^19^. In the same year, Li Hanyu and their team introduced a novel curved-fiber compound-eye camera (CFCEC), employing coherent fiber bundles as the light relay system to transmit sub-images in a curved manner. The CFCEC is equipped with 106 small apertures, offering a total field of view of $160^\circ \times 160^\circ$ and exhibiting advantages such as uniform spacing and slight edge blind spots. A series of experiments were conducted to evaluate the prototype’s field of view, contrast, resolution, and field of view overlap rate. The results demonstrated that the CFCEC has broad development prospects in panoramic monitoring and motion tracking fields^20^. Towards the end of 2023, Wu Qi and their team further optimized their previously proposed camera system (HeCECam) to enhance its detection performance. This optimization included utilizing the “Three-Gravity Subdivision (TGS)” to improve the uniform distribution of sub-eyes and employing a proposed tilt compensation method to enhance compatibility between heterogeneous compound eyes and optical relay systems. Through a series of comparative experiments, the optimized prototype exhibited improved resolution and a wider field of view, enabling HeCECam to deliver better performance in applications such as wide-area monitoring, early warning, and navigation^21^.

Although micro-lens compound-eye cameras have attracted attention due to their compact size and wide field of view, they still have some drawbacks, including low image quality, limited focal length, limited detection distance, and high detection accuracy requirements^22^. Many similar micro-lens array cameras highlight their ability to observe large fields of view at close range and are selectively used for specific applications. Furthermore, most of the compound-eye cameras proposed above are visible light cameras, which are difficult to meet the requirements of all-day detection and have limited detection distances. Therefore, we specifically chose cryogenic infrared detectors. Compared to visible light, the diffraction spot in the mid-wave infrared band we selected is about 10 times larger, making diffraction effects more pronounced. We designed a larger effective aperture, emphasizing the characteristics of large-area, sensitive, and long-range detection of cryogenic mid-wave infrared compound-eye imaging systems. We hope that these systems can play a significant role in the fields of security and military.

## Results

### Optical design of a compound eye imaging system

Drawing from the unique advantages of insect compound eye structures, we have designed an infrared compound-eye optical system that matches cryogenic mid-wave infrared detectors, aiming to achieve passive detection and rapid localization of targets in large field-of-view areas. The choice of cryogenic mid-wave infrared detectors is to minimize the impact of thermal noise. When designing the optical system, it is necessary to simultaneously convert the curved focal surface to a flat focal plane and achieve 100% efficiency of the cold stop. Therefore, the aperture stop of the infrared relay optical system needs to be positioned at the location of the detector’s dewar cold stop to ensure equal size and consistent positioning^23^. Due to the fixed exit pupil position, the relay optical system adopts a two-stage imaging structure to match and connect with the small lens array. The first stage consists of 61 infrared sub-lens arrays on the front spherical surface, and the second stage is the relay optical system that matches the lens array and the cryogenic detector. The design of the infrared sub-lens array is similar to that of traditional single-aperture infrared cameras, where the 61 small apertures can independently image. The relay infrared optical system mainly solves the connection problem between the spherical infrared lens array and the focal plane of the cryogenic infrared detector. Light passes through the spherical infrared lens array to form images, and the spherical images formed by the shell lens array are further imaged on the focal plane of the cryogenic infrared detector through the relay optical system, forming untreated raw scene images. The overall optical system design has a total field distortion of less than 6%^23^.

#### Spherical lens array

**Figure 2:**
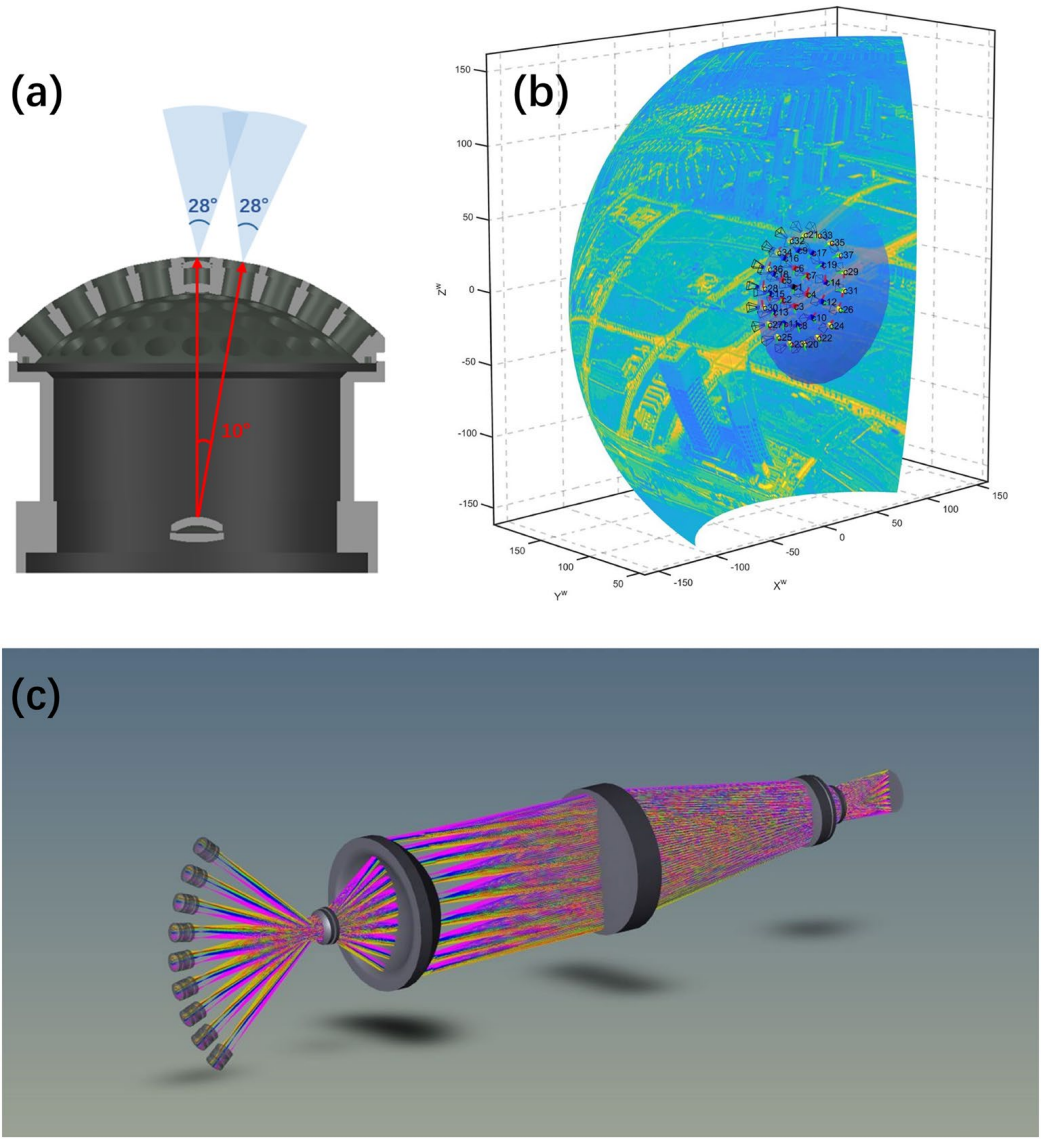
Overall optical design of compound eye camera. (**a**) Sectional view of spherical shell structure. (**b**) Schematic diagram of the infrared sub-lens array imaging of the primary optical system. (**c**) Optical path diagram of the overall optical design of the compound eye camera.

A first-stage imaging system for compound eye infrared cameras. The infrared small lens array is located on a concentric sphere, and the optical axes of all the small lenses intersect at the spherical center of the spherical shell, each small lens system can be imaged independently. The aggregation of the optical axes of the 61 ommatidia of different apertures makes the scene in the field of view of all the ommatidia also located on a concentric sphere, forming a curved image surface, as shown in Fig. 2b. There is some overlap between the lenses of the ommatidia, which slightly improves the *SNR* (signal-to-noise ratio) of the image. Considering the energy transmittance in the infrared band and the feasibility of infrared optical material processing technology, the system constructs a multi-aperture compound eye by using a curved surface arrangement of infrared small lens arrays. The focal length of each ommatidium is 10 mm, the field of view is $28^\circ$, the outer diameter of the ommatidium is 8 mm. Each infrared ommatidium consists of three small lenses, and the optical materials are conventional infrared optical materials such as silicon, zinc sulfide, chalcogenide glass, etc^23^. Nine ommatidia are distributed at the spherical equatorial position. The angle of the field of view of a single ommatidium and the angle of the optical axis between the neighboring ones at the spherical equatorial position are shown in Fig. 2a.

#### Relay optical system

The second-level imaging system of the compound-eye infrared camera. The primary function of the relay optical system is to reimage the curved image formed by the small lens array of the compound eye, transforming the curved focal surface into a flat focal plane and projecting it onto the image plane of the infrared detector, as shown in Fig. 2c. In this mapping process, the relay optical system of the compound eye inevitably generates large-field curvature and large-field aberrations. Generally, the aberrations of the relay optical system in the visible light range of the compound eye can be corrected by adjusting the aperture position to correct large-field curvature and aberrations and adopting a symmetric optical path structure to further eliminate aberrations. However, for the selected cryogenic mid-wave infrared detector, its aperture position is fixed at the end of the optical path, making it impossible to adjust the aperture position, and other methods are needed to correct large-field curvature and other aberrations.

The geometric shape of the spherical surface itself determines the magnitude of the field curvature. For optical systems with large apertures and large fields of view, in addition to the influence of field curvature on the entire system, spherical aberration, and astigmatism also have a significant impact on the image quality of the system. This system optimizes large-field aberrations by introducing two high-order aspheric elements and changing the geometric surface of the lens. To correct large-field curvature, astigmatism, vertical chromatic aberration, and other aberrations, the relay optical system introduces two thick lenses to adjust field curvature and distortion, achieving chromatic aberration balance. The exit pupil of the system coincides with the cold stop of the detector, matching in size and achieving 100% efficiency of the cold stop^23^.

**Figure 3:**
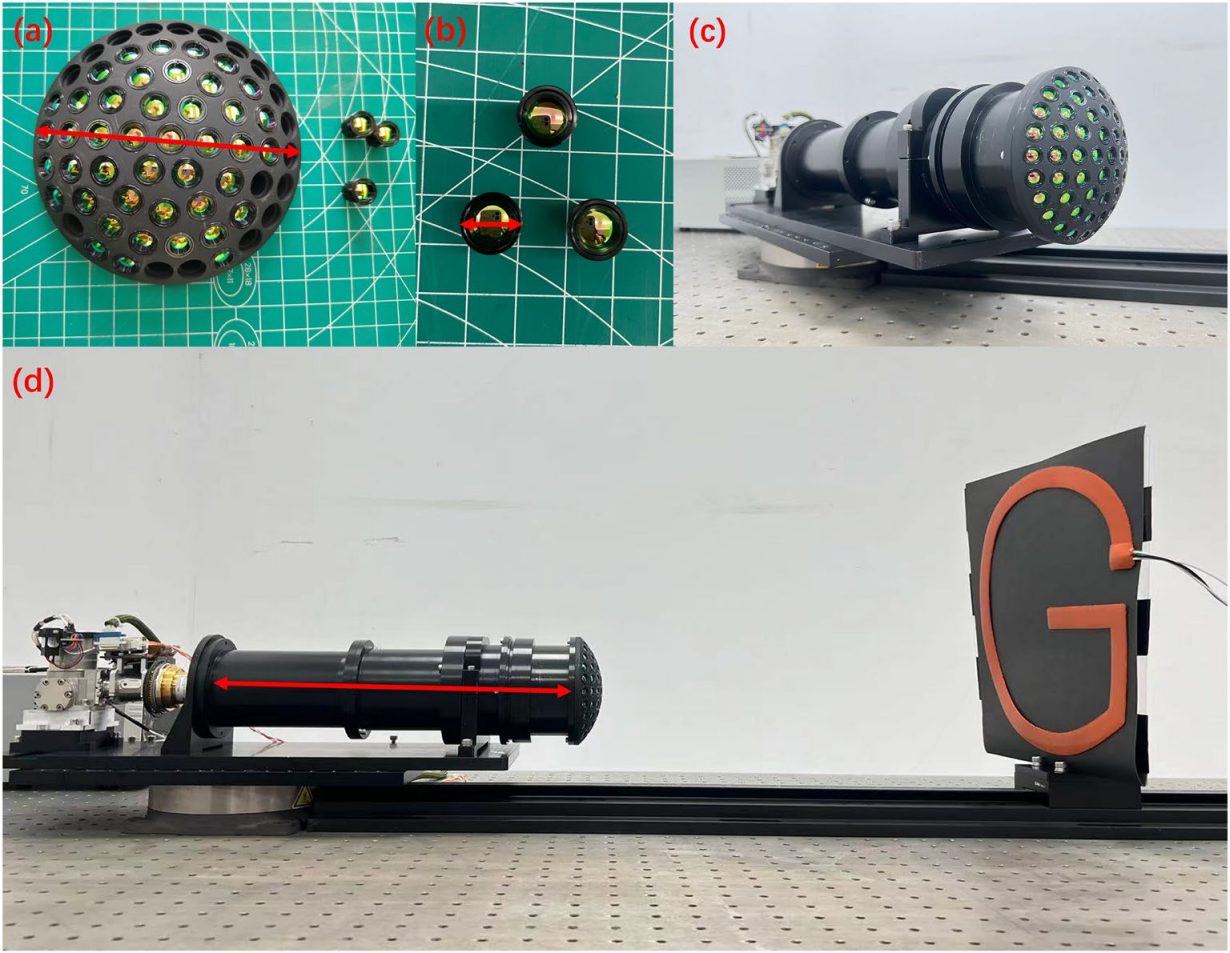
Infrared compound eye camera experiment assembly diagram. (**a**) Curved spherical shell suter diameter = 110 mm. (**b**) Lens outer diameter = 8 mm. (**d**) The length of the relay imaging system =300 mm. Height and width of G = 300 mm. Width of the rubber heating strip = 20 mm.

According to the overall design concept, we have finally developed a complete infrared biomimetic compound-eye camera. The assembly process and setup of the test bench are shown in Fig. 3. The theoretical calculation of the field of view of the designed camera is $108^\circ \times 108^\circ$, as seen in Eq. (1). an image plane size of $1024\times 1024$, and the complete parameter indexes are shown in Table 1. This infrared optical system images the scene in the field of view onto a focal surface with a clear image in each ommatidium. We use an entire optical system to realize multi-aperture imaging simultaneously. Compared to traditional single-aperture infrared optical imaging systems, this system improves the *SNR* to a certain extent. Additionally, it avoids the complex multi-camera alignment connection of traditional separate multi-aperture cameras, reduces the field-of-view error between reconstructed images, and greatly reduces the amount of computation required. The multi-aperture imaging mode enables the acquisition of more scene information. It is expected to calculate target depth information from a single-frame image.

**Table 1:** Infrared compound eye camera machine parameters.

Parameter	Parameter value
Band range	3.7–4.8 $\upmu$m
Temperature range	− 20–60$\,\,{^\circ }\hbox {C}$
NETD(Noise equivalent temperature difference)	32.23 mK
Image size	$1024\times 1024$ pixels
Pixel size	$15\,\upmu$m
Optical system Fno	2
Effective aperture of ommatidium	5 mm
Focal distance of ommatidium	10 mm
Number of ommatidium	61
Total system field of value	$108^\circ \times 108^\circ$
Single ommatidium receiving angle	$28^\circ$
The included angle of the optical axis of adjacent ommatidium	$10^\circ$

## Discussion

**Figure 4:**
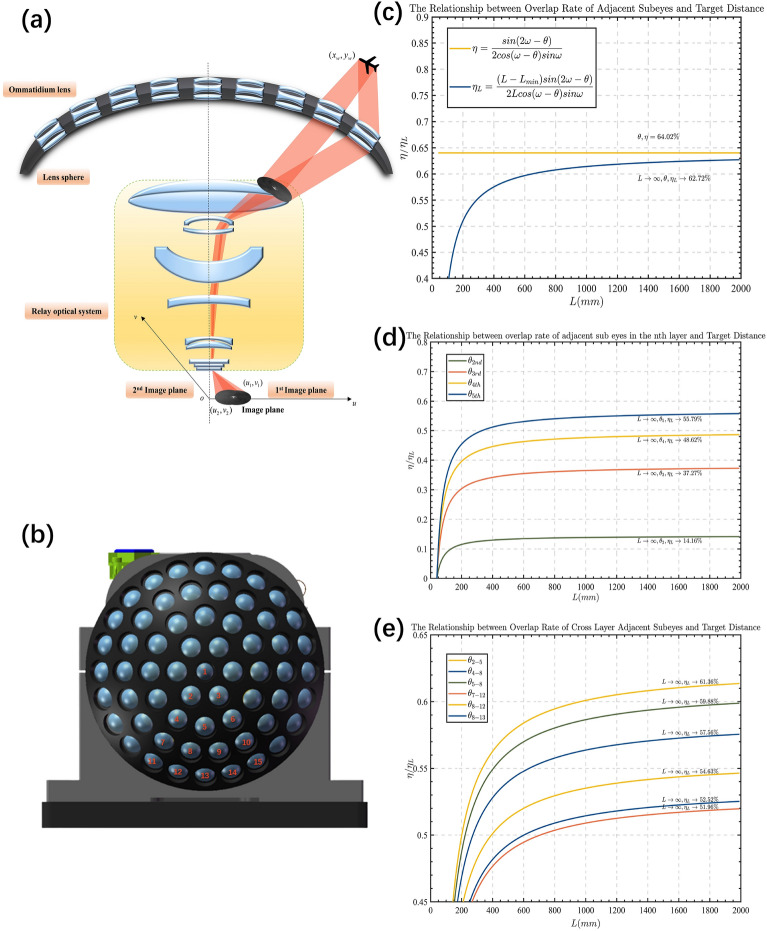
Spherical shell ommatidium position distribution and its field of view overlap ratio. (**a**) Imaging schematic. (**b**) Spherical shell sub-lens array a confident schematic distribution. (**c**) Sub-lens overlap for an optical axis angle of $10^\circ$. (**d**) Overlap ratio of adjacent sub-lenses in each layer. (**e**) Overlap ratio of adjacent sub-lenses across layers.

The primary advantage of compound-eye cameras lies in addressing the limitations of visible light observation due to environmental interference and restricted observation distances, as well as compensating for edge distortion in imaging by single-aperture wide-field cameras. The choice of a cryogenic mid-wave infrared detector is aimed at minimizing the impact of thermal noise, requiring the optical system to achieve 100% efficiency of the cold stop while simultaneously transforming the curved focal plane into a flat one. the schematic diagram illustrating the internal design of the overall optical system is depicted in Fig. 4a. The complex optical design significantly reduces field distortion and edge distortion of compound-eye cameras, achieving an overall field distortion of less than 6%. Additionally, the overlap of certain areas in the field of view improves the *SNR* of imaging, thereby enhancing the target detection capability of infrared compound-eye cameras to some extent. Therefore, the characteristics of large-field detection, passive reconnaissance, reduced edge distortion, rapid response, and other features of infrared compound-eye cameras make them of significant research value in military and security fields, with the reconstruction effect of large-field scenes being crucial.

However, there are still some shortcomings in the imaging performance of compound-eye cameras based on mid-wave infrared detectors. In the results displayed in the experiments, the energy loss in the central ommatidium field is minimal. It can be observed from the experimental results that the imaging energy intensity of the mid-wave infrared compound-eye camera decreases with the hierarchical level of ommatidia from the center toward the periphery. This is partly due to differences in the relative position relationship between targets and different observing ommatidia. Another factor is the special spherical lens of compound-eye cameras, which makes it difficult to achieve consistent light flux for each pixel during energy calibration, especially when multiple layers of lens groups are installed. Traditional calibration based solely on detectors cannot fully compensate for the light flux loss in each ommatidium. This necessitates further research in calibration algorithms. Based on the current conditions, we have proposed a scene reconstruction algorithm leveraging the camera’s special structure, which helps achieve real-time target positioning in infrared compound-eye cameras.

## Methods

### Overlap rate of neighboring ommatidia

The compound eye camera structure is designed in a way that naturally results in some degree of overlap in the field of view between neighboring ommatidia. By determining the ommatidium-axis angle, we can ensure that this overlap rate tends towards a fixed value. We conducted a statistical analysis of the overlap rate between all neighboring ommatidia for the proposed compound eye structure, and the results are presented in Table 2. Our compound eye spherical shell structure comprises 61 ommatidia arranged radially from the center of the spherical shell. These ommatidia are organized in a 5-layer structure. We have calculated the overlap rate between the ommatidia in two categories: overlap across layers and overlap between ommatidia of the same layer. Figure 4c–e demonstrates that the angle of the ommatidium axial pinch is fixed, and its overlap rate stabilizes. The overlapping region of the field of view ensures that no part of the field of view is omitted, resulting in improved energy reception and subsequently enhancing the *SNR* of the system. Furthermore, it provides a matching benchmark for the reconstruction of the large field of view.

Theoretical observation field of view:

1 $$\begin{aligned} FOV_{total} = 2(n_{total}-1)\times \theta + 2\omega  \end{aligned}$$

The overlap rate $\eta$ of adjacent ommatidia is calculated as follows:

2 $$\begin{aligned} \eta = \frac{sin(2\omega -\theta )}{2cos(\omega -\theta )sin\omega } \end{aligned}$$

where $\omega = 14^\circ$, the value of $\theta$ is related to the position of ommatidia, *n* represents the layer of the ommatidium, with the central ommatidium being the first layer arranged radially. The value of $n_{total}$ represents the number of eyes located at the equatorial position of the shell. Due to the central symmetry of the lens’s physical position, the 61 ommatidia can be classified into 8 spatial position relationships, divided into two categories. The first category includes the relationship between adjacent ommatidia in each layer except for the central ommatidium, and $\theta$ for this category can be calculated using Eq. (3).The second category can be summarized as the relationship between adjacent ommatidia across layers in terms of the angle of the optical axis. This includes the clear relationship between 9 ommatidia evenly distributed along the large circular arc of the spherical shell ($\theta =10^\circ$), as well as the relationships between special positions of ommatidia such as 2–5, 4–8, 5–8, 7–12, 8–12, and 8–13. See Fig. 4b for the numbering of ommatidia in the spherical shell. The optical axis angle of these specific positions is obtained through spatial calculations, which can calculate the overlap rate of the field of view, and the neighboring ommatidia optical axis angle equation (4) at specific positions is summarized according to the method of finding the vector angle.

The angle between adjacent ommatidia optical axes on the same layer:

3 $$\begin{aligned} \theta _n = arccos\bigg [1-sin^2(n-1)\theta \cdot \Big (1-cos\big (\frac{\pi }{3(n-1)}\big )\Big )\bigg ], \quad n= 2,3,4,5 \end{aligned}$$

Adjacent layer special position ommatidia’s optical axis angle:

4 $$\begin{aligned} \begin{aligned} \theta _{2-5}&= arccos(cos2\theta cos\theta + cos \frac{\pi }{6}sin2\theta sin\theta ) \\ \theta _{4-8}&= arccos(cos3\theta cos2\theta + cos \frac{\pi }{9}sin3\theta sin2\theta ) \\ \theta _{5-8}&= arccos(cos3\theta cos2\theta + cos \frac{\pi }{18}sin3\theta sin2\theta ) \\ \theta _{7-12}&= arccos(cos4\theta cos3\theta + cos \frac{\pi }{12}sin4\theta sin3\theta ) \\ \theta _{8-12}&= arccos(cos4\theta cos3\theta + cos \frac{\pi }{36}sin4\theta sin3\theta ) \\ \theta _{8-13}&= arccos(cos4\theta cos3\theta + cos \frac{\pi }{18}sin4\theta sin3\theta )\\ \end{aligned} \end{aligned}$$

**Table 2:** Statistics on the overlap rate of adjacent ommatidia.

Relationship between the position of the ommatidia	Cross-Layer adjacent ommatidia		Adjacent ommatidia in the same layer
	Optical axis angle $10^\circ$	Ommatidia in special positions	
Overlap rate ($L \rightarrow \infty$)	$\eta _{1-2}=62.72\%$	$\eta _{2nd}=14.16\%$	$\eta _{2-5}=54.63\%$ $\eta _{4-8}=52.52\%$
	$\eta _{2-4}=62.72\%$	$\eta _{3rd}=37.27\%$	$\eta _{5-8}=59.88\%$
	$\eta _{4-7}=62.72\%$	$\eta _{4th}=48.62\%$	$\eta _{7-12}=51.96\%$
	$\eta _{7-11}=62.72\%$	$\eta _{5th}=55.79\%$	$\eta _{8-12}=61.36\%$
			$$\eta _{8-13}=57.56\%$$

### Energy correction based on compound eye optical system

We chose the Stirling-cooled mid-wave infrared detector because its energy response error is relatively small, even with ambient temperature changes and temperature drift. After conducting comprehensive tests on various correction algorithms, we selected a commonly used one-point correction and improved integration time adjustment based on the two-point correction algorithm. This was used in conjunction with eliminating the nonuniformity of the pixel response to eliminate the detector’s original image in the blind element. The principle is shown in Eqs. (5)–(8), where (*i*, *j*) in $S_{i,j}\left( \phi \right)$are the coordinates of the detector cells in the image array, $g_{i,j}\left( \phi \right)$ is the pixel response rate, $\Phi$ is the irradiance incident to the detector cells, $o_{i,j}\left( \phi \right)$ is the dark current; and $\phi _1$ is the calibration point to obtain the average difference $D_{i,j}$ to compensate for the image element’s dark current. The basic two-point correction selects two radiance calibration points, $\phi _L$ and $\phi _H$ for correction. The experiments compare the non-uniform correction effects of one-point correction and integral time-adjusted two-point correction algorithms in various scenarios.

A one-point correction compensates for the dark current:

5 $$\begin{aligned} \overline{S}(\phi _1)= & {} \frac{1}{N \times M}\sum _{i=1}^N\sum _{j=1}^MS_{i,j}(\phi _1) \end{aligned}$$

6 $$\begin{aligned} D_{i,j}= & {} S_{i,j}(\phi _1) - \overline{S}(\phi _1) \end{aligned}$$

The two-point correction corrects the gain factor:

7 $$\begin{aligned} \overline{S}_L= & {} \frac{1}{N \times M}\sum _{i=1}^N\sum _{j=1}^MS_{i,j}(\phi _L) \end{aligned}$$

8 $$\begin{aligned} \overline{S}_H= & {} \frac{1}{N \times M}\sum _{i=1}^N\sum _{j=1}^MS_{i,j}(\phi _H) \end{aligned}$$

**Figure 5:**
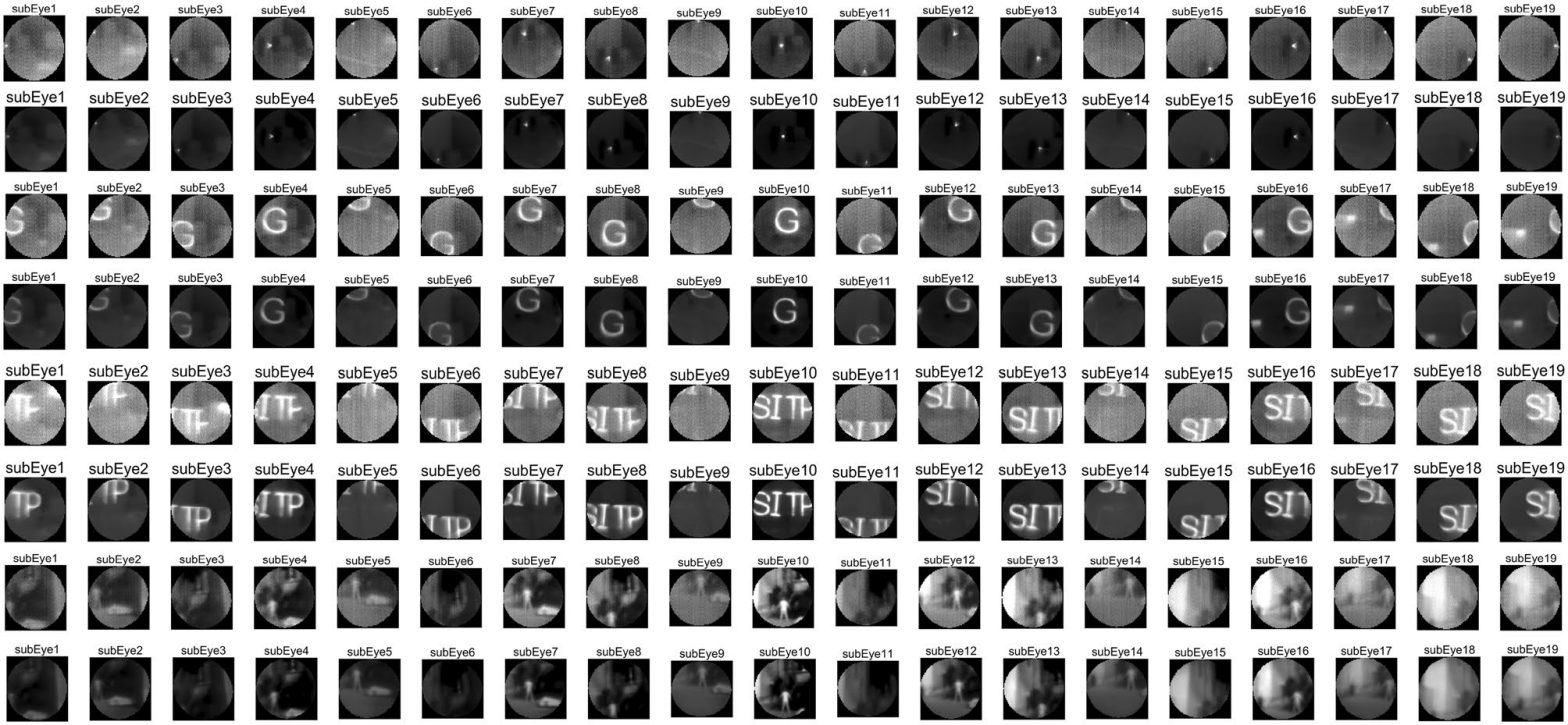
Comparison of calibration results for point light, single letter, multi-letter and scene images.

The straight lines determined by $(\phi _L,{\overline{S}}_L)$ and $(\phi _H,{\overline{S}}_H)$ is used as the calibration line, and the two-point calibration based on integration time takes into account the influence of different integration times on the detector’s pixel response curve. By collecting batches of data points at different response times, a better correction of the gain coefficient is achieved. The compound-eye camera system for a single detector with multiple apertures can perform the correction for all ommatidia simultaneously, and the correction results for different scenes are depicted in Fig. 5.

**Figure 6:**
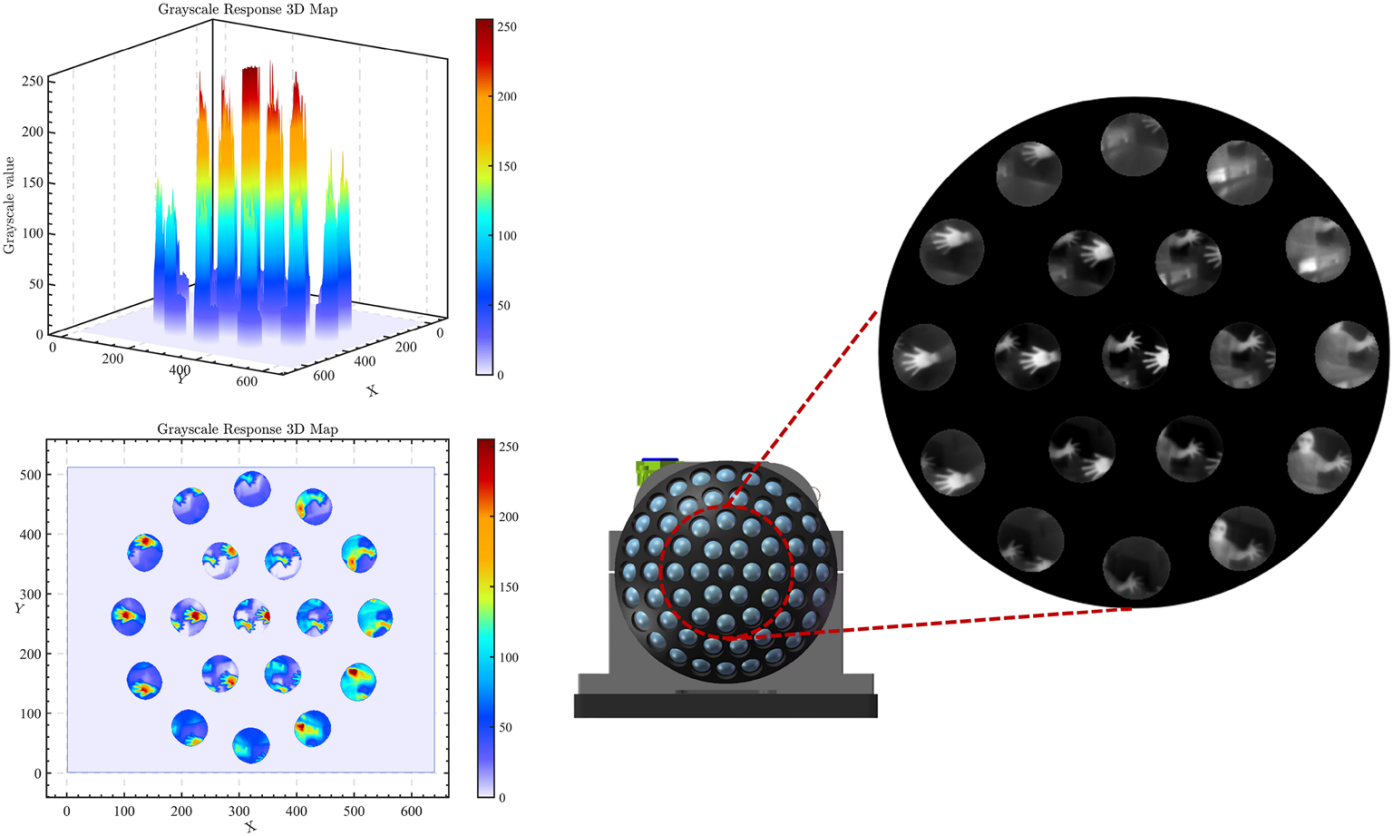
Gray-scale response of each ommatidium.

Experiments show that the generalized one-point and two-point correction algorithms are still insufficient for correcting compound eye images, see Fig. 6. Due to the special structure of the compound eye camera, the angle between the optical axis of the small lenses causes the outer ring of ommatidia to experience energy attenuation in their projection. Due to the small angle between the optical axis of the ommatidia, we will approximate attenuation as a linear function to compensate for the energy. $I_{true}$ represents the true value of the grayscale image whereas $I_{redu}$ represents the image’s attenuation. The enhancement coefficients use $\tau$ and the equation is shown in (9):

9 $$\begin{aligned} I_{true} = \tau \cdot I_{redu} \end{aligned}$$

The calculation of $\tau$ is based on statistical data obtained by analyzing a large number of experimental results, which compensates for the energy attenuation to a certain extent, but it is still necessary to further explore the energy correction method for the special compound eye camera structure.

**Figure 7:**
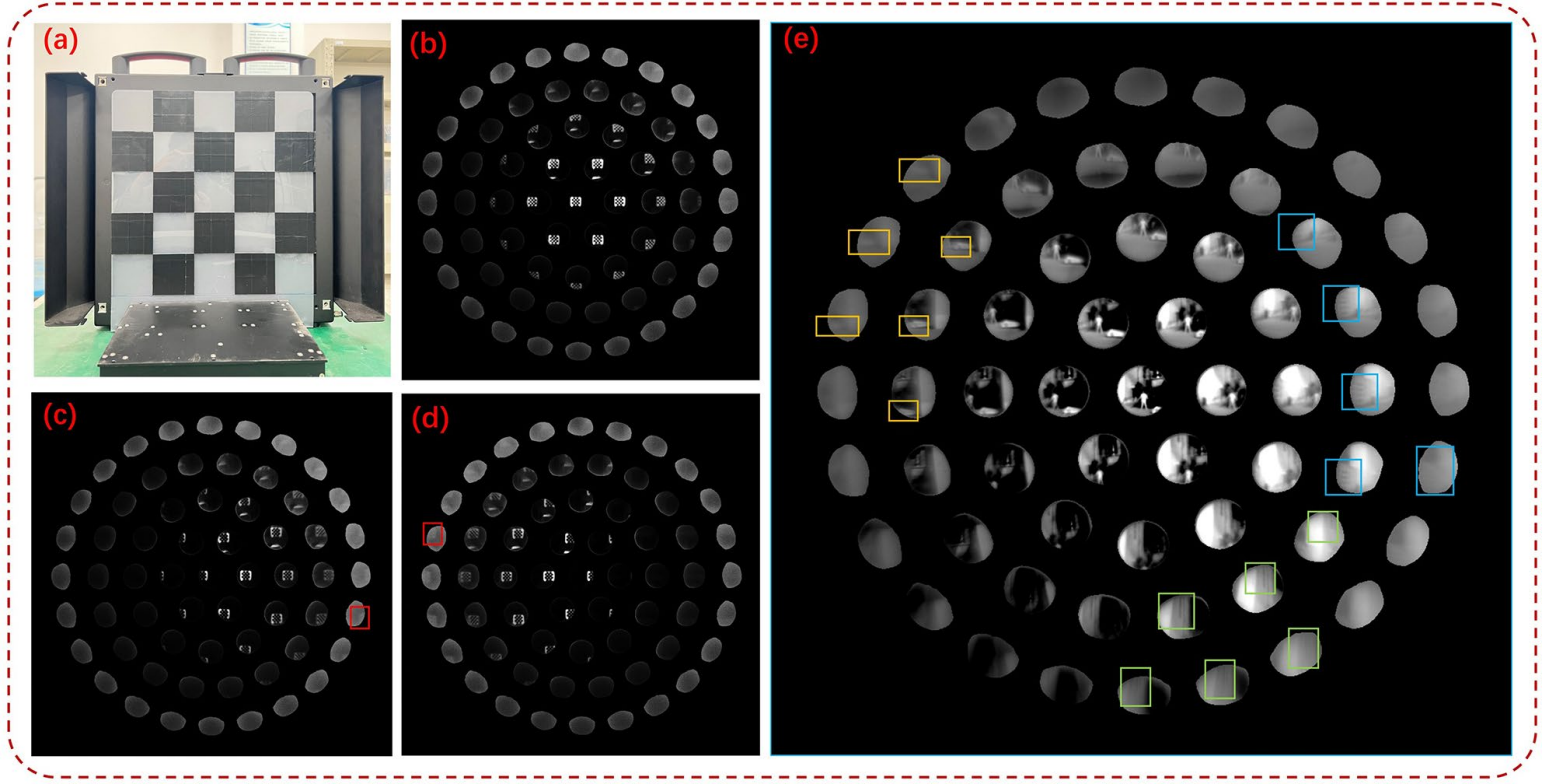
Imaging results of infrared compound eye camera system. (**a**) Checkerboard target homemade. The red markings indicate the positions of checkerboards in the fifth layer of ommatidia. (**b**), (**c**) and (**d**) Imaging effect of target at different angles. (**e**) The outdoor imaging effect of a compound eye camera. The yellow markings indicate the position of ground containers, the green markings indicate the words of the building, and the blue markings represent rectangular buildings.

Figure 7 displays the laboratory and outdoor scene images that were directly captured. The panoramic image allows the ommatidia at the edge of the fifth circle to detect changes in optical flow of moving targets, but the image detail quality is poor. As previously mentioned, errors were encountered during manual assembly, and specific calibration methods for the spherical infrared camera were lacking, resulting in a progressive loss of energy in the compound eye camera’s ommatidia. The advantage of small edge distortions is evident in the compound eye structure camera. The distortion of ommatidia in the compound eye structure is significantly reduced under the same field of view, resulting in smaller edge distortions in the reconstructed image. The theoretical field of view range of the camera was tested by calibrating it on a parallel light pipe calibration platform against strong target sources.

### Overlapping pixel fusion stitching of neighboring ommatidia reconstructs the large field of view

**Figure 8:**
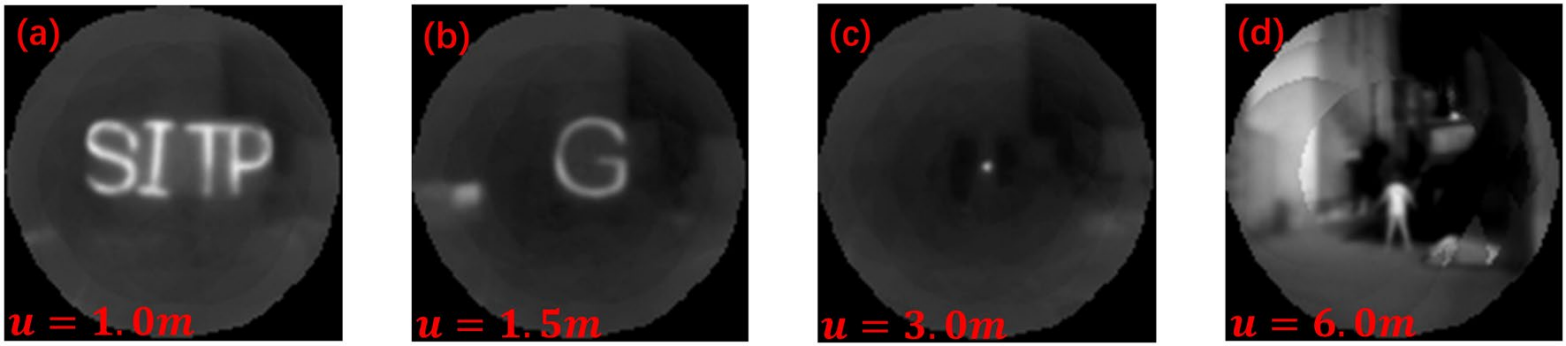
19 ommatidia stitching results. *u* represents the distance of the object. (**a**) Height = 300 mm, width $\approx$ 600 mm. Heating strip width =20 mm. (**b**) Height and width = 300 mm. Heating strip width = 20 mm. (**c**) Diameter halogen light source = 10 mm. (**d**) Human height = 1.6 m.

The basic steps of the traditional image stitching algorithm include detection, alignment, and stitching of feature points. This algorithm is commonly used to deal with images that have certain overlapping regions. The stitching algorithm has been developed to be relatively mature and effective so far. On one hand, due to the constraints of the detection field of view and image resolution, as well as the fact that the actual imaging pixels of the ommatidium we designed are fewer and the field of view is blurred, the actual detected feature points are not enough to support the stitching algorithm. On the other hand, the compound-eye camera is designed to serve for the observation of a large field of view and rapid target localization, and the traditional stitching method is extremely arithmetic-intensive and time-consuming. Based on the above two reasons, the traditional stitching method cannot be applied to single-detector compound-eye structure cameras, and we propose an ommatidium information extraction and fixed-pixel alignment stitching method for engineering applications. There are some errors in information extraction for pixels at the edge of the ommatidium, and we use the distance of the pixel from the center of the image plane as a confidence metric to classify the small lens imaging into confidence levels. The advantage of the complex optical design is that it can directly project the curved surface scene as a flat image, and the relationship between each ommatidium scene is a two-dimensional transformed position, which greatly reduces the computational complexity and improves the accuracy of real-time detection. The spatial position transformation is calculated using the inter-ommatidium visual field overlap rate $\eta$ as the stitching prior knowledge, the two-dimensional direction vectors of the 19 ommatidia centers concerning the center of the imaging plane are calculated $E=e_1,e_2,\ldots ,e_{19}$. The relative displacement matrices $M=m_1,m_2,\ldots ,m_{19}$, where *m* has a dimension of $2\times 2$, and which contains information about positional offsets of the two-dimensional pixels. Divide the 19 ommatidia’s pixel point locations as $L_1,L_2,\ldots ,L_{19}$ (where the pixel size of each layer of ommatidia is not the same, according to its layer is divided into $n_1,n_2,n_3$, the dimension of $L_1$ is $n_1\times 2$, the dimension of $L_2-L_7$ is $n_2\times 2$. The dimension of $L_8-L_{19}$ is $n_3\times 2$). The positional transformation of each ommatidium image when stitching is shown in Eq. (10). To ensure imaging uniformity, the field-of-view energy response was retained for the overlapping part based on its trust level, the center ommatidium scene in the orthopic field condition had the highest trust level. The compound eye camera stitched the 19 ommatidia in the orthopic field to reconstruct the following scene, see Fig. 8.

10 $$\begin{aligned} L^s= & {} L\cdot M \cdot E \end{aligned}$$

11 $$\begin{aligned} L_i^s= & {} L_i\cdot m_i \cdot e_i, \quad i=1,2,\ldots ,19 \end{aligned}$$

Combined with the overlap rate between neighboring ommatidia mentioned in the previous section, the image *SNR* of the infrared compound eye camera in different scenes is statistically calculated. The theoretical derivation is based on single-frame detection, assuming that the probability distribution of the noise satisfies the Gaussian distribution as shown in Eq. (12). The threshold segmentation threshold is taken as $T_h = \mu +k\sigma$, where *k* is the threshold coefficient. The detection probability of the target pixel and the detection probability of the background pixel can be regarded as the detection probability $P_d$ and the false alarm probability $P_f$, respectively, as in Eqs. (13) and (14). The relationship between the detection probability and the false alarm rate of a single frame can be deduced, as in Eq. (15). It can be seen that the higher the *SNR*, the higher the detection rate, while the false alarm rate will decrease and the detection distance will be longer.

The experiment selects the sub-image *SNR* of the seven ommatidia in the center with the highest overlap rate of the field of view for statistics; In the actual calculation, $I_{signal}$ is the image signal variance, $I_{noise}$ is the noise variance, and the numerical results of the table are the field-of-view scene *SNR* after stitching and the sub-image scene *SNR* of each sub-image, as shown in Table 3. The experiments show that there is a certain degree of improvement in the *SNR* of the sub-images of the compound eye camera after stitching.

Assumed noise probability distribution:

12 $$\begin{aligned} p(n)=\frac{1}{\sqrt{2\pi }\sigma }exp\big [-\frac{(n-\mu )^2}{2\theta ^2}\big ] \end{aligned}$$

Detection probability $P_d$ and false alarm probability $P_f$:

13 $$\begin{aligned} p_d= & {} 1-\phi \left( \frac{T_h-\mu }{\sigma }-SNR\right) \end{aligned}$$

14 $$\begin{aligned} p_f= & {} 1-\phi \big (\frac{T_h-\mu }{\sigma }\big ) \end{aligned}$$

Relationship between detection probability $P_d$ and false alarm probability $P_f$:

15 $$\begin{aligned} \phi ^{-1}(P_d)-\phi ^{-1}(P_f)=SNR \end{aligned}$$

Image *SNR* calculation:

16 $$\begin{aligned} SNR=20\cdot log_{10}\big (\frac{I_{signal}}{I_{noise}}\big ) \end{aligned}$$

**Table 3:** *SNR* of single-sub-image vs. multi-sub-image stitching *SNR*.

	Center ommatidium	1	2	3	4	5	6	7 sub-images stitching *SNR*	19 sub-images stitching *SNR*
**Point source**	0.8720	1.2886	1.4540	1.3292	1.6708	1.6320	1.6729	1.6575	**1.9906**
**Mono-letter**	0.7217	1.0860	1.1542	1.1323	1.1885	1.1867	1.1252	1.1274	**1.5348**
**Multi-letter**	1.1412	1.3447	1.3348	1.4015	1.3627	1.4192	1.3549	1.3466	**1.7456**
**Scene**	1.3562	1.1366	1.3349	1.3387	1.4586	1.3740	1.5911	1.5744	**1.6377**

## Data Availability

The datasets generated and/or analyzed during the current study are not publicly available due to our laboratory’s confidentiality agreement, but are available from the corresponding author upon reasonable request.

## Code availability

The codes used for panoramic imaging are available from the main developer (wangxy991@mail.ustc.edu.cn) upon reasonable request.
